# Self-assembled nanosheets of biocompatible polymers as universal cell-membrane mimic to block viral infection

**DOI:** 10.1016/j.bioactmat.2025.06.035

**Published:** 2025-07-03

**Authors:** Ranen Etouki, Na Xing, Mathias Dimde, Chuanxiong Nie, Ievgen S. Donskyi, Kai Ludwig, Ehsan Mohammadifar, Rainer Haag

**Affiliations:** a Institut für Chemie und Biochemie, Freie Universität Berlin, Takustr. 3, 14195, Berlin, Germany; b Forschungszentrum für Elektronenmikroskopie und Gerätezentrum BioSupraMol, Institut für Chemie und Biochemie, Freie Universität Berlin, Fabeckstr. 36A, 14195 Berlin, Germany

**Keywords:** Supramolecular structures, Broad spectrum antiviral materials, Nanosheets, Influenza a virus inhibitor, Herpes-simplex virus inhibitor, Virucidal inhibitor

## Abstract

Viruses cause severe damage to society due to seasonal and pandemic outbreaks; therefore, developing new antivirals is urgently needed. Multivalent virus inhibitors are promising broad-spectrum antivirals, as they can block the initial step of viral infection by mimicking the structure of the cell receptors on the host cell membrane. Biocompatible supramolecular architectures are particularly well-suited for virus inhibition due to the numerous weak non-covalent bindings, resulting in strong yet dynamic multivalent interactions. Herein, we report on supramolecular nanosheets based on dendritic polyglycerol (dPG). The dPG core was functionalized with different ratios of sulfate and mercaptoundecanoic acid (MUA) groups. The MUA, as the hydrophobic part, triggers the self-assembly and -via the acid group-the supramolecular interaction with the virus, while sulfate groups mimic heparan sulfate proteoglycans (HSPG) on the cell membrane for virus interaction. The effect of polymer functionalization degree of MUA (ranging from 30 to 100 %) on the nanosheet size and morphology, as well as their interaction with viral particles, were monitored by cryo-transmission electron microscopy (cryo-TEM) and cryo-electron tomography (cryo-ET). Bio-functional assays such as plaque reduction, pre-infection inhibition, hemagglutination inhibition (HAI) and cell viability assays have been performed to assess the *in vitro* efficiency of supramolecular nanosheets against Influenza A virus and Herpes-simplex virus type 1. These studies revealed inhibitory activities against IAV (X31/H3N2) and HSV-1 with the half-inhibitory concentration (IC_50_) of 1 and 0.01 μg/mL *in vitro*, respectively, demonstrating its potential of being a universal virus inhibitor by dynamic multivalent interactions.

## Highlights

• The functionalized dendritic polyglycerols self-assemble in supramolecular nanosheets.
• The supramolecular nanosheets are active against Influenza A and Herpes-simplex virus type 1 with an IC_50_ value of 1 μg/mL.
• Supramolecular nanosheets showed virucidal activity against Herpes-simplex virus type 1.
• The binding of Nanosheets to Influenza A virus particles are visualized by cryo-electron microscopy.

## Graphical abstract

**Figure:**
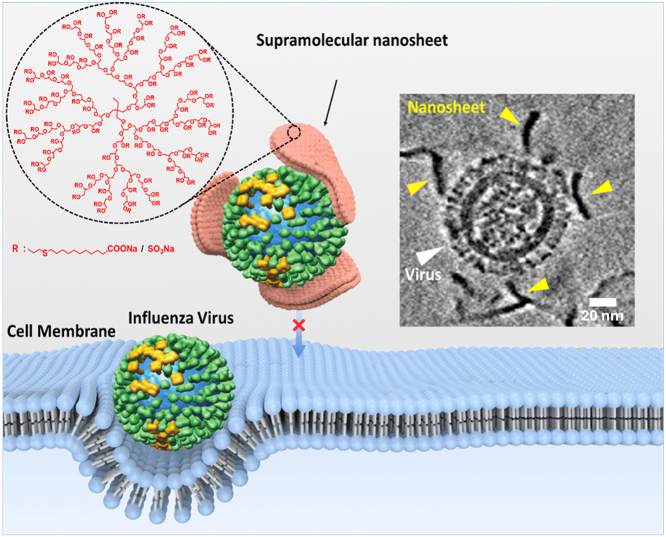

## 1 Introduction

Viral infections can rapidly escalate into pandemics due to their ability to spread quickly among human populations [[1], [2], [3]]. New viruses can appear or re-emerge every 3–5 years [4], and their rapid spread facilitates the emergence of mutant virus variants, as recently seen with SARS-CoV-2 and previously influenza A and B viruses [[5], [6], [7]]. Although vaccines and traditional medicinal drugs are effective in mitigating the impact of many viral diseases, they are only able to act against specific viruses, following the “one bug−one drug” concept. Therefore, there is an urgent need for broad-spectrum antivirals capable of targeting a wide range of existing and emerging viruses regardless of their antigenic evolution. These antivirals should usually bind competitively to the viral envelope or its surface proteins in a multivalent manner, thus preventing virus-cell interactions, which is typically the initial and common step of infection for many types of viruses [2,8]. This concept led to the discovery of heparin as the first broad-spectrum antiviral drug in the 1960s which was active against several viruses [9]. Heparin mimics the heparan sulfate proteoglycan (HSPG), a molecule expressed on the surface of almost all eukaryotic cell types, which is a binding target for numerous viruses such as human immunodeficiency virus type 1 (HIV-1), herpes simplex virus (HSV), human cytomegalovirus (HCMV), human papillomavirus (HPV), human respiratory syncytial virus (RSV), SARS CoV-2 and flaviviruses [[10], [11], [12]]. Previous studies have shown that HSPG-mimicking materials such as sulfated polysaccharides, polymers, dendrimers, and nanoparticles are potent antivirals against a wide range of enveloped viruses [[13], [14], [15]]. Negatively charged sulfated groups are likely to competitively bind to positively charged amino acid residues on virus particles through electrostatic interaction, thereby shielding the surface proteins of the virus and ultimately reducing its infectivity [[16], [17], [18], [19]].

In a partially similar approach, certain viruses, such as SARS-CoV-2 and influenza A virus, have been shown to interact with self-assembled materials through supramolecular interactions [20,21]. Prior to COVID-19, all recent respiratory infection pandemics were caused by animal-origin influenza A viruses (IAVs) that crossed the species barrier to infect humans. The surface of influenza A virus is coated with the receptor-binding protein hemagglutinin (HA) and the receptor-cleaving enzyme neuraminidase (NA), which work together to regulate the interactions between the virus and the host cell [22,23]. The interaction of influenza virus with host cells and other bioactive materials occurs through a multivalent mechanism, involving numerous weak and dynamic interactions that are thought to originate from supramolecular interactions [[24], [25], [26]]. Some previous researches have shown the ability of supramolecular nanostructures in sensing as well as blocking the cell infection by influenza virus [20,27,28]. These results indicate supramolecular inhibitors as interesting antivirals to be further investigated. However, despite the extensive list of materials demonstrating antiviral efficacy *in vitro*, none have shown efficacy in humans. Only a few have progressed to clinical trials, with some failing and others still under investigation [1,29,30].

One possible reason for the suboptimal effect of the drugs is the fact that the virustatic nature of the inhibitor is based on the reversible binding to the virus. Dissociation of inhibitor in an equilibrated manner after dilution in body fluids leads to the release of active virus. Therefore, developing of viral inhibitors that are able to stablish strong interactions with viruses, so that they either remain bound even after dilution or damage the viral envelope, can lead to a more effective drug [1,12]. In recent years, researchers have demonstrated that incorporation of hydrophobic moieties in the chemical structure of viral inhibitors not only enhances their efficacy in preventing viral infections but also ensures that their inhibitory effects remain effective even after dilution. This class of compounds is referred to as virucidal inhibitors [[31], [32], [33], [34]]. In our previous research, we synthesized a virus inhibitor based on dendritic polyglycerol (dPG) which self-assembles in well-defined and monodisperse nanosheets under physiological conditions capable of binding to SARS-CoV-2 and inhibiting its infectivity in a virucidal way [21]. dPG has been widely used as a suitable platform for biomedical applications due to properties such as high water solubility, ease of synthesis, soft and globular structure and the facile functionalization with other ligands of interest [[35], [36], [37]]. In this work, we design a broad-spectrum antiviral platform based on dPG backbone which is decorated with sulfate and mercaptoundecanoic acid (MUA) groups. Due to the hydrophobicity, MUA triggers self-assembly in nanosheet structure in aqueous medium. We hypothesize that the multivalent supramolecular nanosheet interacts with the virus envelope and its proteins through supramolecular interactions, while the sulfate groups increase the interaction with HSPG-dependent viruses. In addition, the long hydrophobic chains results in high affinity and maintain an irreversible inhibition mechanism.

## 2 Materials and methods

### 2.1 Synthesis of functionalized dPG macromolecules

Firstly, dPG-allyl with different degree of functionalization (DF) was synthesized (eq. corresponding to different DF are summarized in Table S1). dPG (1.1 g, 10 kDa, 13.5 mmol hydroxyl groups) was dried at 60 °C under high vacuum overnight and dissolved in DMF followed by addition of NaH and stirring for 30 min. The reaction was cooled down with an ice bath. Allyl bromide was dissolved in DMF and added dropwise to the cooled reaction. The suspension was allowed to stir at 40 °C for 24 h. The reaction was quenched by addition of methanol and purified by dialysis against methanol for 3 d. All dPG-allyl compounds were obtained as a colourless and viscous product with 30 %, 50 %, 80 % and 100 % DF.

Coupling of MUA. General thiol-ene click reaction procedure for all DF (equivalents for all reactions and further details are summarized in Table S2). dPG-allyl was dissolved in methanol and MUA was added. The stirred reaction was purged with nitrogen for 15 min to remove oxygen from the solution. Then DMPA and TCEP (catalytical amount) were added, and the reaction was irradiated with UV light (λ = 365 nm) at room temperature for 6 h. The functionalized polymers were purified by dialysis against MeOH/CHCl_3_ for 3 d. After solvent removal under reduced pressure, all dPG-C were obtained as white solids with DF of 30 %, 50 %, 80 % and 100 % named as dPG-C_0.3_, dPG-C_0.5_, dPG-C_0.8_ and dPG-C respectively. Half of the polymers were kept for the sulfation step and the rest was dissolved in water by addition of 1 M NaOH followed by dialysis to remove the remaining NaOH solution.

Sulfate functionalization of remaining OH groups. The remaining hydroxyl groups of dPG-C_0.3_, dPG-C_0.5_, dPG-C_0.8_ were converted to sulfate group by reaction with sulfur trioxide complex (Py•SO_3_). The equivalents for all reactions and further details are summarized in Table S4 dPG-C was dissolved in DMF, and Py•SO_3_ dissolved in DMF was added dropwise to the stirred solution at 60 °C. The reaction was allowed to stir at 60 °C for 24 h and then quenched with water. The pH was adjusted to 8 by adding 1 M NaOH solution. The resulting reaction mixture was first dialyzed against brine solution and subsequently against brine with decreasing NaCl concentration over 2 d and finally against water for additional 2 d. After lyophilization dPG-C_0.3_S_0.7_, dPG-C_0.5_S_0.5_, dPG-C_0.8_S_0.2_ were obtained as white solids.

### 2.2 Characterization of samples

#### 2.2.1 Cryo-transmission electron microscopy (cryo-TEM)

Perforated carbon film-covered microscopical 200 mesh grids (R1/4 batch of Quantifoil, MicroTools GmbH, Jena, Germany) were cleaned with chloroform and hydrophilized by 60 s glow discharging at 8 W in a BALTEC MED 020 device (Leica Microsystems, Wetzlar, Germany) before 4 μl aliquots of the corresponding solution were applied to the grids. The samples were vitrified by automatic blotting and plunge freezing with a FEI Vitrobot Mark IV (Thermo Fisher Scientific Inc., Waltham, Massachusetts, USA) using liquid ethane as cryogen. The climate in the chamber was set at 22 °C and 100 % humidity. The grids with the vitrified specimens were assembled to so-called autogrid cartridges and transferred to the autoloader of a FEI TALOS® ARCTICA electron microscope (Thermo Fisher Scientific Inc., Waltham, Massachusetts, USA). This microscope is equipped with a high-brightness field-emission gun (X-FEG) operated at an acceleration voltage of 200 kV. Micrographs were acquired on a FEI Falcon 3 EC direct electron detector (Thermo Fisher Scientific Inc., Waltham, Massachusetts, USA) at a calibrated primary magnification of 28,000x using the microscopes low-dose protocol.

#### 2.2.2 Cryo-electron tomography (cryo-ET)

To obtain spatial information of the samples, tomograms were recorded on the TALOS® ARCTICA transmission electron microscope (ThermoFisher Scientific Inc., Waltham (MA), USA) at 200 kV. For this purpose, single axis tilt series (±64° in 2° tilt angle increments) were acquired with a FEI Falcon 3 EC 4k × 4k direct electron detector using a Volta Phase Plate at 28 K primary magnification with a total dose lower than 120 e−/Å2. Reconstruction of the tomograms was performed with binned data (binning factor 2) using ThermoFisher Inspect3D software, version 4.5.2. The increased contrast provided by the phase plate facilitated the alignment of the individual tilt images, but also slightly reduced the transmission of high spatial frequencies. The 3D voltex presentation was prepared using Amira, Version 6.2 (ThermoFisher Scientific Inc., Waltham (MA), USA).

### 2.3 In vitro assays

All biological assays were performed in biological triplicates over the course of 3 different days. All cell lines were cultured in Dulbeccos Modified Eagle Medium (DMEM) with 10 % fetal bovine serum (FBS) and 1 % penicillin/streptomycin (P/S) and incubated at 37 °C and 5 % CO_2_.

#### 2.3.1 Cellular toxicity with CCK8

The cellular toxicity was determined using Cell Counting Kit 8 (CCK8) from Hycultec GmbH (Beutelsbach, Germany) against MDCKII dog kidney epithelial cells, Vero E6 African green monkey kidney epithelial cells and HBE human bronchial epithelial cells. Cells were seeded in 96 well-plates and incubated overnight. The compounds were dissolved in PBS to the concentration of 1 mg/mL and diluted 10-foldly in duplicates and added to the cells. Untreated cells and SDS (1 %) acted as positive and negative controls, respectively. The cells were incubated with the materials for 45 min at 37 °C and then treated with 10 % CCK8 in DMEM. Using a micro-plate reader, absorbance was then measured at a wavelength of 450 nm. The cellular toxicity was calculated by normalizing the absorbance of the untreated cells to 100 % viability and relating absorbance of treated cells accordingly. The results were visualized with Origin 2024b.

#### 2.3.2 Herpes-simplex virus 1

Vero E6 cells were used for plaque reduction, pre-infection inhibition, virucidal assays and HSV-1 propagation. HSV-1 viruses utilized in this study were derived from F strain pYEbac102 and were kindly provided by Dr. Y. Kawaguchi [38], University of Tokyo, Japan. A cytomegalovirus immediate early promoter-driven green fluorescent protein (GFP) marker within the bacterial artificial chromo-some (BAC) mini-F backbone was used for detection upon reconstitution.

##### 2.3.2.1 Pre-infection inhibition assay

Vero E6 cells were seeded in 96 well plates. Samples were serially diluted 10-fold (ranging from 0.1 μg/mL to 1 mg/mL) and then added to the cells. After being incubated for 45 min at 37 °C/5 % CO_2_, the cells were infected with HSV-1 with a multiplicity of infection (MOI) of 0.1 for 48 h at 37 °C/5 % CO_2_. Then, cells were washed with PBS, the nuclei were stained with Hoechst 33342 (1 μg/mL) for 30 min, washed with PBS and fixed with formaldehyde (4 %) for 30 min. Fluorescent images were acquired using a ZEISS Axio Observer 7 (Apotome.2) microscope (ZEISS, Germany).

##### 2.3.2.2 Plaque reduction assay

The serially 10-fold diluted samples were incubated with 100 plaque forming units (PFU) for 45 min at room temperature. Then, the mixture of sample and virus was added to the confluent Vero E6 cells and incubated for another 45 min. Subsequently, unbound virions were washed with PBS, and the cells were cultured with Avicel overlay medium for 3–4 days for plaque development. Then, cells were washed with PBS, fixed with formaldehyde (4 %) for 30 min and stained with crystal violet. The percentage of plaque reduction was calculated as follows:

$$\text{Plaque}\;\text{reduction}\;\left[\%\right]=\left(1-\frac{\text{Plaque}\;\text{number}\;\left(\text{sample}\right)}{\text{Plaque}\;\text{number}\;\left(\text{virus}\;\text{control}\right)}\right)\times 100\%$$

The half-maximal inhibitory concentration (IC_50_) values were estimated using GraphPad Prism 6 utilizing dose-response inhibition fitting.

##### 2.3.2.3 Virucidal assay

An effective concentration (1 mg/mL) of samples were incubated with HSV-1 (6 x 10^5^ PFU/mL) diluted in DMEM for 45 min at rt. A 10-fold dilution series of this mixture was added on cells and incubated for 45 min at rt. Afterward the inoculum was removed, cells were washed and incubated with Avicel overlay medium for 3–4 days at 37 °C. Then, cells were washed with PBS, fixed with formaldehyde (4 %) for 30 min and stained with crystal violet. The viral titer was calculated based on the plaque numbers and the dilution factor. The results were visualized using GraphPad Prism 6.

#### 2.3.3 Influenza A virus (IAV)

MDCK II cells were used for pre-infection inhibition and IAV propagation. The IAV strain utilized in this study was X-31 (subtype H3N2).

##### 2.3.3.1 Pre-infection infection assay

MDCK II cells were seeded in 24 well plates. Samples were diluted 10-foldly with 100 μL and incubated with 100 μL of IAV (strain X-31) with an MOI of 1 for 45 min. Then, this mixture was added to the cells and incubated for 45 min at rt. Subsequently, the cells were washed with PBS and incubated with infection medium (MEM, without FBS or trypsin) at 37 °C for 24 h. Then, cells were fixed with formaldehyde (4 %) for 30 min, IAV was detected with the FITC-conjugated antibody (mouse anti flu A NP, MA1-7322, Invitrogen) and the nuclei was stained with Hoechst 33342 (1 μg/mL) for 30 min. Fluorescent images were acquired using a ZEISS Axio Observer 7 (Apotome.2) microscope (ZEISS, Germany).

##### 2.3.3.2 Hemagglutinin inhibition assay (HAI assay)

Samples were diluted in duplicates in 2-fold with 25 μL in a U-shaped well-plate. IAV was diluted to 8 hemagglutination units (HAU)/50 μL and 25 μL were added to the diluted samples. The sample virus mixtures were incubated at rt with continuous shaking for 30 min. Then, 50 μL of 1 % red blood cells (RBC) were added to each well and incubated at rt for 1 h.

## 3 Results

### 3.1 Synthesis and characterization of polymers

**Fig. 1:**
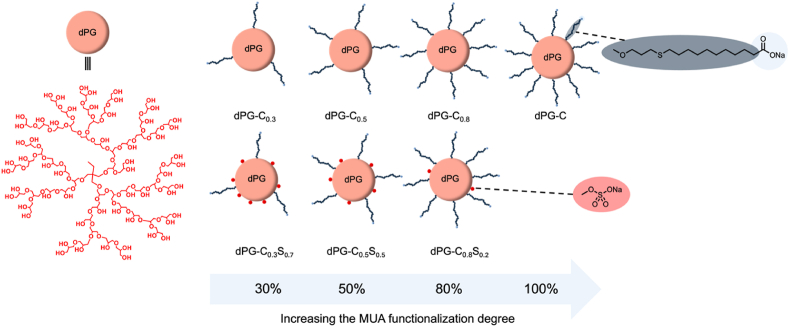
Schematic structure of functionalized dendritic polyglycerols with different MUA a sulfate functionalization degree.

**Fig. 2:**
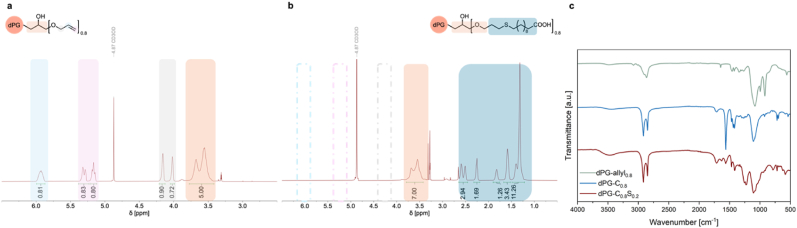
(a) ^1^H NMR spectrum and signal assignment for dPG-allyl_0.3_. DF were determined by correlating the allyl protons with the polyglycerol backbone protons. ^1^H NMR data for other materials are presented in Figure S2 (b)^1^H NMR spectrum and signal assignment of dPG-C_0.8._ All allylic groups were converted to carboxylic moieties, as indicated by disappearance of the allylic protons signals and the appearance of upfield signals corresponding to the protons of the ligand. ^1^H NMR data for other materials are presented in Fifure S4 (c) FTIR spectra of dPG-allyl_0.8_, dPG-C_0.8_, and dPG-C_0.8_ S_0.2_. FTIR data for other materials are presented in Fig. S6.

To develop broad-spectrum antiviral materials, we synthesized a library of dPG-based supramolecular materials incorporating MUA and sulfate groups with different degree of functionalization. The presence of both sulfate and carboxyl groups contribute to a synergetic effect in the interaction between the viruses and the 2D self-assembly. The carboxylic groups of the MUA facilitate the self-assembly of dPG into a sheet-like structure with large surface area, thereby enabling virus wrapping via electrostatic interaction and increasing the multivalency. In addition, the sulfate groups as HSPG mimicking moiety are incorporated into the sheets, enabling the inhibition of viruses which use HSPG to enter the cell. MUA (C11) was utilized for functionalization since our previous work [20] demonstrated no self-assembly when dPG was functionalized with alkyl chains with below C11. Moreover, alkyl chains with >C9 can irreversibly disrupt the viral membrane [31,39]. Toward this aim, the hydroxyl groups of the dPG core were functionalized with different functionalization degrees of MUA (30, 50, 80 and 100 %) while the rest of hydroxyls were converted to sulfate groups. The different DF were selected to systematically investigate the impact of different DF, ensuring a comprehensive analysis across a broad range of modification levels. These specific values were chosen to cover a representative spectrum from low to complete functionalization, allowing an analysis of potential trends, structure relationships and threshold effect. dPG with 10 kDa molecular weight and hydrodynamic volume of 5 nm which present around 130 terminal hydroxyl groups have been used. Fig. 1 shows a schematic presentation of the chemical structure of the synthesized polymers. The synthesis was performed with the functionalization of dPG as the core through the allylation reaction of terminal hydroxyl groups to serve as a suitable platform for further click reaction. The DF has been measured by ^1^H NMR analysis of the dPG-allyl, by integrating the allyl protons (5.3-5.1 and 6.0–5.8 ppm) with the dPG backbone protons (3.7–3.2 ppm) (Figs. 2a and S2). In the next step an UV-initiated thiol-ene click reaction was applied to enable efficient and selective conjugation of MUA ligands to dPG-allyl by using DMPA as the photo initiator (Fig. S3). This approach was chosen due to mild reaction conditions, high specificity and rapid reaction kinetics under ambient conditions [40,41]. The success of the end-group functionalization was confirmed through elemental analysis and ^1^H NMR spectroscopy. Elemental analysis revealed the incorporation of the desired MUA ligand, with the experimentally determined carbon, hydrogen and sulfur content aligning with the theoretical values predicted based on the expected structure (Table S3). In addition, the ^1^H NMR spectra of the dPG-MUA product exhibited distinct proton signals (1.1–2.7 ppm) corresponding to the alkyl chain of MUA. Disappearance of the allylic proton signals is another proof of successful thiol-ene click reaction (Figs. 2b and S4). By using the same reaction procedure with determined equivalent of allyl bromide and further click reaction with MUA four distinct materials, characterized by 30 %, 50 %, 80 % and 100 % of degree of functionalization were synthesized and named as dPG-C_0.3_, dPG-C_0.5_, dPG-C_0.8_, and dPG-C (Fig. 1). Next, the remaining hydroxyl groups of all polymers were converted to sulfate groups by sulfur trioxide pyridine complex for the controlled introduction of anionic sulfate groups to enhance the water solubility and surface interaction with different glycoproteins of different HSPG-binding viruses, making the materials more suitable for subsequent applications as a broad virus inhibitor [17,42]. Since the hydroxyl groups were converted to sulfate groups the polymers after sulfation are named as dPG-C_0.3_S_0.7_, dPG-C_0.5_S_0.5_, dPG-C_0.8_S_0.2_. Elemental analysis verified successful conversion of hydroxyl groups to sulfate groups via an observed increase in sulfur content (Table S5). Zeta-potential and size were measured and it was determined that all compounds exhibit negative charges and the structures remained well-dispersed in buffered solution (Tables S6 and S7, Fig. S7).

### 3.2 Cryo-TEM measurements of the self-assembled nanosheets

**Fig. 3:**
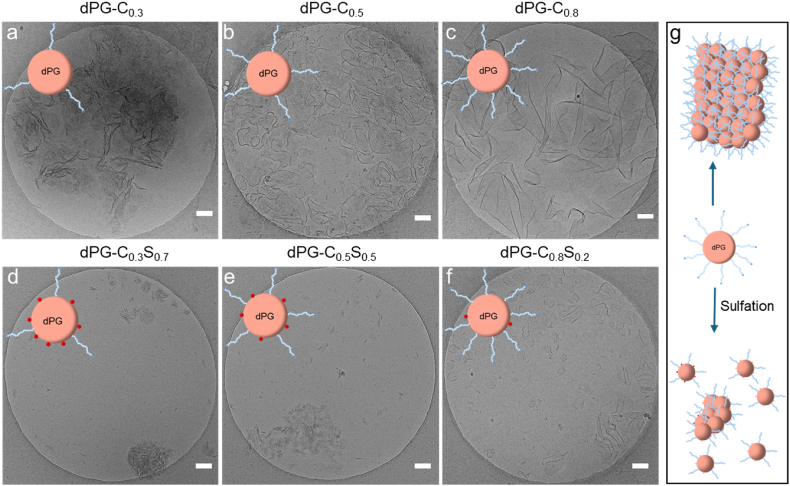
Cryo-TEM images of 1 mg/mL polymer solution in PBS (pH 7.4 and salt concentration 152.7 mM). The functionalized dPG macromonomers self-assemble in ultra-thin nanosheets. The incorporation of sulfate groups in the polymer backbone results in the formation of small size nanosheets or individual isolated dPG macromonomers. The scale bars corresponding to 100 nm.

Due to the presence of alkyl chains, which enhance the hydrophobicity of the polymers, and carboxylic acid groups, which facilitate hydrogen bonding within the system, we measured the polymer structure in a phosphate buffer saline solution with a salt concentration of 152.7 mM and a pH of 7.4. The detailed mechanism of nanosheet formation has been discussed in our previous study (Mohammadifar et al., 2025) [21] when dPG is fully functionalized with MUA, it forms well-defined supramolecular nanosheets in aqueous solutions as the nanosheet structure is the most stable architecture for dPG-MUA amphiphiles as they have an ellipsoidal structure. The mechanism of the supramolecular sheet formation of our materials is governed by a balance between attractive and repulsive forces. The attractive forces arise from hydrogen bonding between the carboxyl groups and hydrophobic interactions between the aliphatic chains of the polymers. These forces synergistically stabilize the amphiphilic polymer in the nanosheet structure. Whereby the repulsive forces result from the electrostatic repulsion between negatively charged carboxylate groups on the surface of the polymers prevents excessive aggregation or clustering of the polymers. Small changes such as incorporating sulfate groups to the structures can disturb the balance and changes the size of the supramolecular nanostructures [21]. However, to investigate the effect of the degree of functionalization with MUA groups and the incorporation of sulfate groups into the polymer backbone on the size and morphology of the newly synthesized polymers, Cryo-TEM measurements were performed. The Cryo-TEM images showed that all the dPG functionalized with MUA form nanosheets regardless of their degree of functionalization. As can be seen in Fig. 3a–c, the lateral size of the nanosheets increases when the enhancing the degree of functionalization of dPG with MUA. It can also be observed that the folding and wrinkling of nanosheets decreases with increasing amount of MUA. This phenomenon may indicate a reduction in the flexibility of the nanosheets. Such behavior can be attributed to the increased hydrophobicity of the nanosheets. However, the incorporation of sulfate groups into the polymer structure increases the hydrophilicity of the self-assemblies, leading to the formation of nanosheets with smaller lateral size (Fig. 3d–f).

### 3.3 Cellular compatibility

**Fig. 4:**
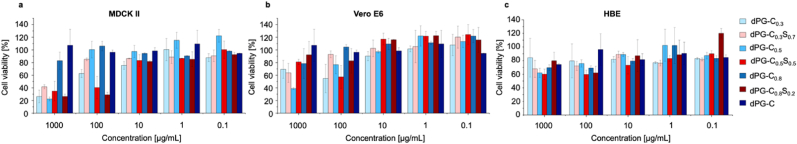
(a) Cell viability against MDCK II cells using CCK8. The data is presented as mean ± SD. (b) Cell viability against Vero E6 cells using CCK8. The data is presented as mean ± SD. (c) Cell viability against HBE cells using CCK8. The data is presented as mean ± SD.

Since the previous studies have shown that supramolecular aggregations can bind to viruses through supramolecular interactions, including H-bonding, hydrophobic, and electrostatic interactions [28,43], and the polymers containing sulfate groups act as attachment receptors for a wide range of viruses [11,44], we decided to evaluate the potential of our materials in IAV and HSV-1. Cell cytotoxicity of the synthesized polymers was first evaluated by cell counting kit-8 (CCK-8) assay. For this aim, MDCK-II and Vero E6 cells were used, as they are the host cells for the propagation of influenza A X31/H3N2 virus and HSV-1, respectively. As shown in Fig. 4 the cell viability assay showed no significant cytotoxicity against the cell line up to a concentration of 1 mg/mL.

### 3.4 In vitro inhibition assay of nanosheets against Influenza A viruses

Then we evaluated the virus inhibitory efficiency against influenza A virus, strain X-31. For this purpose, a pre-infection inhibition assay was conducted to assess viral inhibition. As seen in Fig. 3b all compounds display an antiviral activity with a half maximal inhibitory concentration (IC_50_) of approximately 1 μg/mL. Influenza A virus is not known to interact with sulfate, so we hypothesize that the mechanism behind the IAV inhibition involves the non-specific supramolecular interactions between self-assembled nanosheets and the virus. This can be confirmed by the HAI results (Fig. S8), which indicate that hemagglutination was observed across all tested concentrations (500–0.24 μg/mL). This suggests that the compounds do not bind to HA specifically, and the inhibition of infection is a result of steric shielding by the nanosheets. This is also evidenced by our observation that all the samples showed similar inhibitory activities despite the degree of sulfation. Moreover, the large surface area facilitates efficient virus shielding and minimizing the interaction between viruses and sialic acid moieties on the cell membrane, thereby reducing potential cell entrance and ensuing infection. It should be noted that the self-assembled nanosheets are soft and flexible materials and can adapt to the surface morphology of the viruses for maximized interactions. Regardless of lateral size, the nanosheet formation is the key feature in virus inhibition meaning that the nanosheet-virus interaction is not based on specific receptor-ligand interactions, but the steric shielding/wrapping by the nanosheets.

**Fig. 5:**
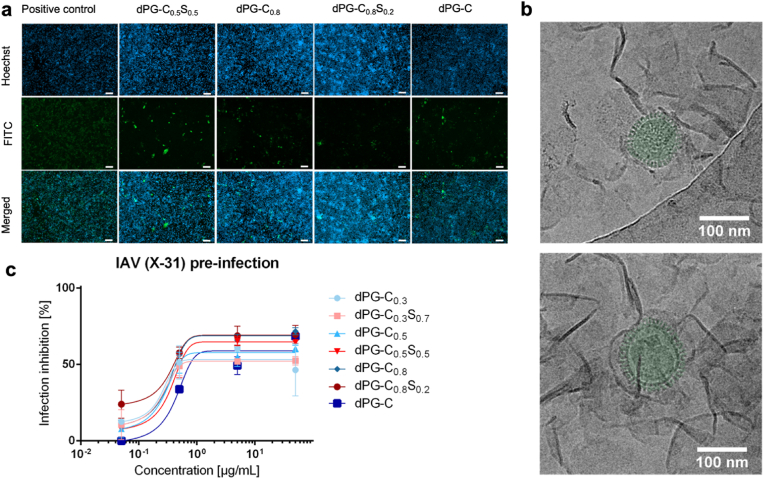
(a) IAV pre-infection assay. Cell nuclei were stained with Hoechst (blue) and infected cells were stained by antibodies with FITC (green). The depicted concentration is 500 μg/mL. The scale bars present 100 μm. Images for all materials are presented in S11. (b) Cryo-TEM images of polymer solution in PBS (1 mg/mL) and IAV (green colored) encapsulated by these supramolecular nanosheets. (c) IAV pre-infection assay against IAV, X-31 strain. Compounds showed antiviral activity with an IC_50_ of approximately 1 μg/mL. The data is presented as mean ± SD. Statistical analysis: All data were expressed in this manuscript as mean ± SD. All the results have been performed three times by independent experiments. A two tailed Student's t-tests was used to analyze the statistical significance between the two groups by using GraphPad Prism 7.0 (GraphPad Software Inc.).

Additionally, the interaction of nanosheets with the influenza virus was visualized by cryo-TEM (Fig. 5b and supplementary video) and cryo-ET after incubation of nanosheets with a virus for 1 h in the PBS solution (Fig. 7). In particular, reconstruction of the 3D volume using cryo-electron tomography revealed that the nanosheets have the potential to literally enclose IAV due to their large surface area, thus preventing binding to the cell membrane and subsequent infection.

### 3.5 In vitro inhibition assay of nanosheets against HSV-1

**Fig. 6:**
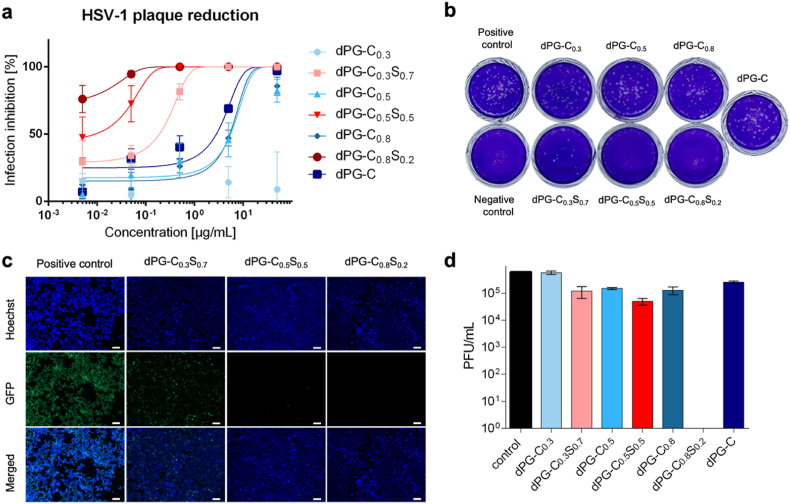
(a) HSV-1 plaque reduction assay. dPG-C_0.5_S_0.5_ and dPG-C_0.8_S_0.2_ reveal IC_50_ values below 0.01 μg/mL. Non sulfated compounds exhibit antiviral activity with an IC_50_ of approximately 10 μg/mL. The data is presented as mean ± SD. Statistical analysis: All data were expressed in this manuscript as mean ± SD. All the results have been performed three times by independent experiments. A two tailed Student's t-tests was used to analyze the statistical significance between the two groups by using GraphPad Prism 7.0 (GraphPad Software Inc.). (b) HSV-1 plaque reduction assay. Cells were stained with crystal violet. The depicted concentration is 0.5 μg/mL (c) HSV-1 pre-infection inhibition assay. Cell nuclei were stained with Hoechst (blue) while infected cells express GFP (green). The depicted concentration is 1 μg/mL. The scale bar presents 100 μm. Images for all materials are presented in S12. (d) Virucidal assay: HSV-1 was incubated with samples or DMEM for 45 min and then serially diluted in 10-fold manner. Dilutions were then added to Vero E6 cells and incubated for 72 h. Results are presented as mean ± SD.

While influenza viruses use sialic acids as their natural receptor, herpes simplex viruses use heparan sulfate [45]. Therefore, in order to have broad-spectrum antivirals we incorporated sulfate groups in the macromolecules and evaluated the inhibition efficacy of the nanosheets on HSV-1. The HSV-1 inhibition tests were conducted using plaque reduction assay (Fig. 6a–b) and pre-infection inhibition assay (Fig. 6c). All the sulfated compounds showed higher HSV-1 inhibitory activities than the non-sulfated counterparts. However, with increasing sulfation degree, we observed a decrease in HSV-1 inhibition as seen in Fig. 6a and b. This could be related to the fact that with decreasing sulfation degree an increase in the size of the nanosheets is observed. This leads to the conclusion that larger nanosheets enhance the efficacy of HSV-1 inhibition due to their shielding ability. This indicated that an appropriate ratio between sulfation degree and lateral size is crucial for optimal inhibition of HSV-1. The highest antiviral activity of dPG-C_0.8_S_0.2_ may also be a result of its higher functionalization of MUA, which leads to irreversible interactions with HSV-1 envelope, which is discussed in the next section.

**Fig. 7:**
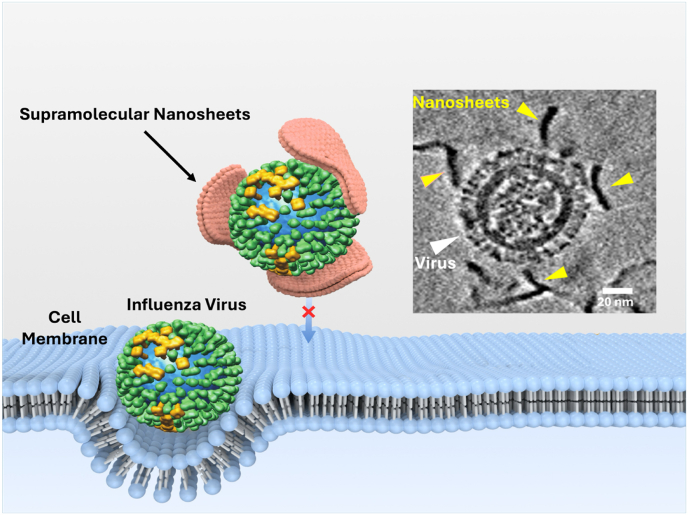
Representation of the interaction of nanosheets with influenza A virions. Top right: A central slice of the reconstructed 3D volume of a cryo-electron tomogram taken after 1 h after incubation of dPG-MUA with influenza virus A/X-31. The nanosheets and virus are indicated by yellow and white triangles respectively. The schematic representation of the interaction of nanosheets with virus, leads to the inhibition of cell entry, is shown in the cartoon section of the figure. The nanosheets are shown in salmon color.

### 3.6 Virucidal assessment of nanosheets against HSV-1

Since the irreversible inhibition mechanism (virucidal) of virus inhibitors is a key feature in progressing to further preclinical studies, we evaluated the virucidality of nanosheets against HSV-1. The results indicated that dPG-C_0.8_S_0.2_ demonstrated significant virucidal activity against HSV-1, reducing the virus titer by 3 orders of magnitude (Fig. 6d). This observation may be due to the fact that dPG-C_0.8_S_0.2_ contains the highest number of hydrophobic MUA chains. However, dPG-C, the compound with only MUA functionalization but no sulfation, did not show any virucidal activities, highlighting the role for sulfation for virus binding. Morphologically, as seen in Fig. 3f, dPG-C_0.8_S_0.2_ forms sheets with a large surface area which implies the presence of a higher number of exposed alkyl chains, which are proven to act in a virucidal mechanism [31,39]. In contrast, dPG-C_0.3_S_0.7_ and dPG-C_0.5_S_0.5_ contain less alkyl chains form smaller nanosheets which aggregate in bigger “rose-like” structures (Fig. 3d and e) and therefore leads to a smaller surface area and thus fewer exposed alkyl chains. In addition, dPG-C_0.5_S_0.5_ and dPG-C_0.8_S_0.2_ indicated virucidal activity as well. However, since dPG-C_0.8_S_0.2_ is a potent inhibitor (IC_50_ < 0.01 μg/mL) against HSV-1, it can effectively inactivate the viruses completely.

## 4 Conclusion

In general, we developed a library of dPG-based macromolecules with different functionalization degree of mercaptoundecanoic acid and sulfate groups. The macromolecules self-assemble in supramolecular nanosheets while the higher amount of sulfate groups result in nanosheets with a smaller lateral size or isolated macromolecules. *In-vitro* experiments revealed that all compounds demonstrated inhibitory activity against influenza virus, with an IC_50_ value of about 1 μg/mL, indicating their potential effectiveness in inhibiting viral cell entry under the tested conditions. In contrast, the results revealed that sulfated compounds were more potent inhibitors (IC_50_ < 0.01 μg/mL) against HSV-1, demonstrating superior antiviral activity compared to the non-sulfated materials. This indicates the crucial role of sulfated groups for the inhibition of HSV-1 and the sheet formation for the inhibition of IAV. While the dPG-C_0.8_S_0.2_ displayed comparable inhibitory activity against IAV, it showed the most significant irreversible (virucidal) activity against HSV-1. These results identifying dPG-C_0.8_S_0.2_ as the most effective candidate for potential preclinical application. Many synthetic polymeric materials have been reported in the literature with antiviral activities with a receptor dependent mechanism which work specifically against only one virus. For example, heparin has been used as antivirals against HSV-1. In addition, synthetic multivalent sialic acid nanostructures show inhibition against influenza. However, these materials act virus specific. The advantage of our system is this supramolecular structure can inhibit virus infection in a receptor-independent approach – steric shielding by wrapping the virus. We believe our study can be inspirative for more designs of active and universal virus inhibitors.

## CRediT authorship contribution statement

**Ranen Etouki:** Writing – review & editing, Writing – original draft, Validation, Investigation. **Na Xing:** Writing – original draft, Supervision, Investigation. **Mathias Dimde:** Writing – original draft, Investigation. **Chuanxiong Nie:** Writing – original draft, Supervision, Investigation. **Ievgen S. Donskyi:** Writing – original draft, Funding acquisition. **Kai Ludwig:** Writing – original draft, Investigation. **Ehsan Mohammadifar:** Writing – original draft, Supervision, Investigation, Conceptualization. **Rainer Haag:** Writing – original draft, Supervision, Resources, Funding acquisition, Conceptualization.

## Ethics approval and Consent to participate

Ethical approval was not required, as the blood samples were commercially obtained and anonymized.

## Declaration of competing interest

The authors declare no conflict of interest.
