# Supplementary material for: Self-assembled nanosheets of biocompatible polymers as universal cell-membrane mimic to block viral infection
Source: Bioact Mater. 2025 Jul 3;52:857–65. doi: 10.1016/j.bioactmat.2025.06.035 (PMC12270697; doi:10.1016/j.bioactmat.2025.06.035)
Supplement: Multimedia component 1 [file mmc1.docx]

**Self-assembled nanosheets of biocompatible polymers as universal cell-membrane mimic to block viral infection**

**Supporting information**

Figure S1. Reaction conditions of the allylation.

| **DF**  **[%]** | **dPG** | | | **NaH**  **(60% dispersion in mineral oil)** | | | **Allyl bromide** | | |
| --- | --- | --- | --- | --- | --- | --- | --- | --- | --- |
|  | m  [g] | n_FG_ [mmol] | eq. | m  [g] | n [mmol] | eq. | m  [g] | n [mmol] | eq. |
| **30** | 1.1 | 13.5 | 1.0 | 0.5 | 13.5 | 1.0 | 0.5 | 4.05 | 0.3 |
| **50** | 1.1 | 13.5 | 1.0 | 0.5 | 13.5 | 1.0 | 0.9 | 7.43 | 0.6 |
| **80** | 1.1 | 13.5 | 1.0 | 0.5 | 13.5 | 1.0 | 1.3 | 1.8 | 0.8 |
| **100** | 1.1 | 13.5 | 1.0 | 1.6 | 40.5 | 3.0 | 3.3 | 27.0 | 2.0 |

Table S1: reaction scales for the allylation reaction.

dPG-C_0.8_

dPG-C


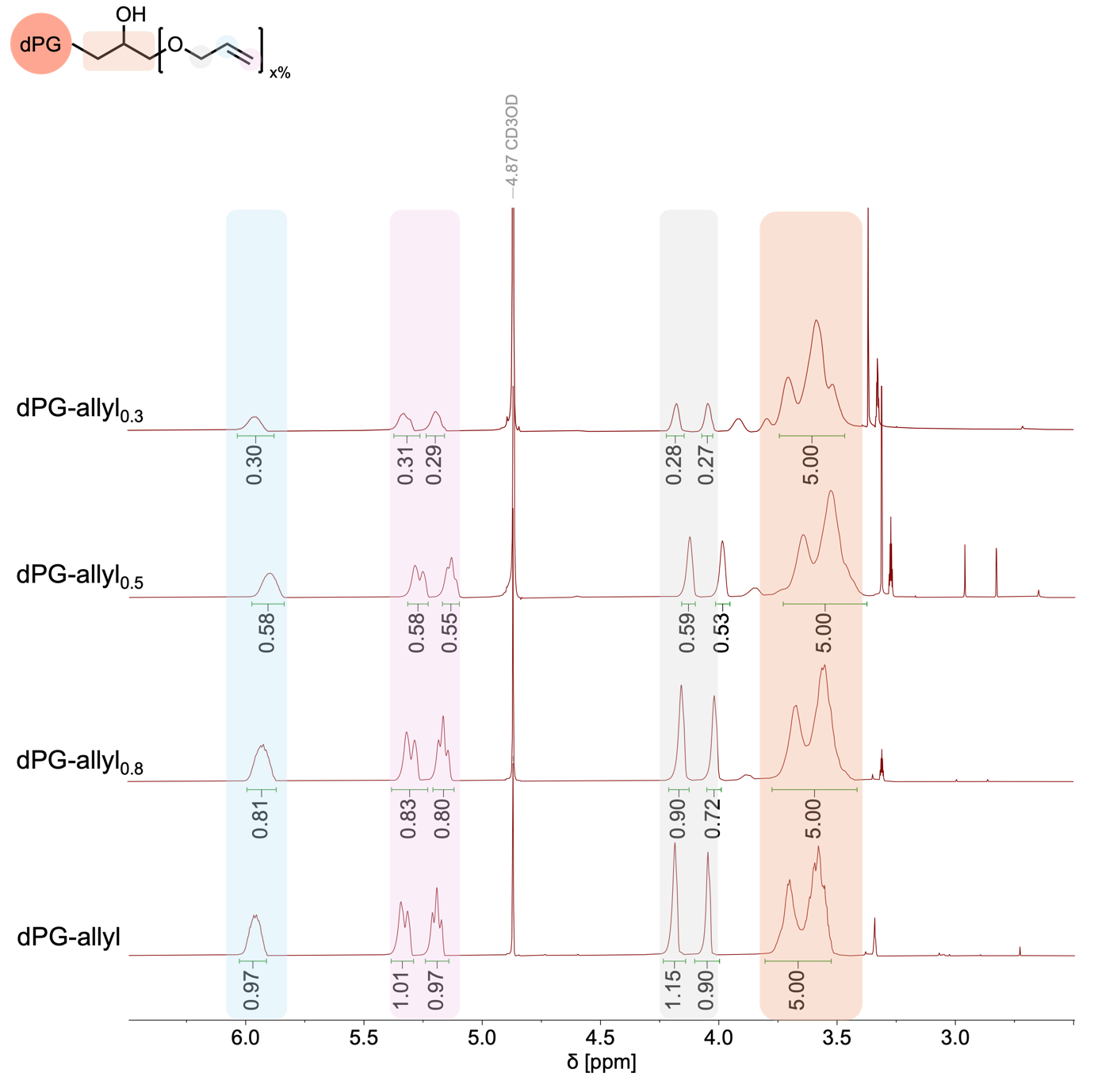


Figure S2. ^1^H-NMR spectra and signal assignment for all allylated materials_._ DF were determined by correlating the allyl protons with the polyglycerol backbone protons.

Figure S3. Reaction conditions of the thiol-ene click reaction.

Table S2: reaction scales for the thiol-ene click reaction.

| **DF**  **[%]** | **dPG-allyl** | | | **MUA** | | | **DMPA** | | |
| --- | --- | --- | --- | --- | --- | --- | --- | --- | --- |
|  | m  [g] | n_FG_ [mmol] | eq. | m  [g] | n [mmol] | eq. | m  [mg] | n [mmol] | eq. |
| **30** | 1.0 | 11.6 | 1.0 | 2.5 | 11.6 | 1.0 | 15.6 | 0.116 | 1 mol% |
| **50** | 1.0 | 10.6 | 1.0 | 2.3 | 10.6 | 1.0 | 14.2 | 0.106 | 1 mol% |
| **80** | 1.0 | 9.4 | 1.0 | 3.1 | 14.1 | 1.5 | 18.9 | 0.141 | 1 mol% |
| **100** | 1.0 | 8.7 | 1.0 | 3.7 | 17.4 | 2.0 | 23.3 | 0.174 | 1 mol% |

Table S3: Elemental analysis of carboxylated polymers.

|  | **C [%]** | **H [%]** | **N [%]** | **S [%]** |
| --- | --- | --- | --- | --- |
| **dPG** | 51.3 | 8.2 | 0.0 | 0.0 |
| **dPG-C_0.3_** | 55.0 | 10.9 | 0.0 | 5.5 |
| **dPG-C_0.5_** | 57.8 | 11.2 | 0.0 | 7.6 |
| **dPG-C_0.8_** | 59.1 | 11.1 | 0.0 | 8.6 |
| **dPG-C** | 61.7 | 10.3 | 0.0 | 11.8 |


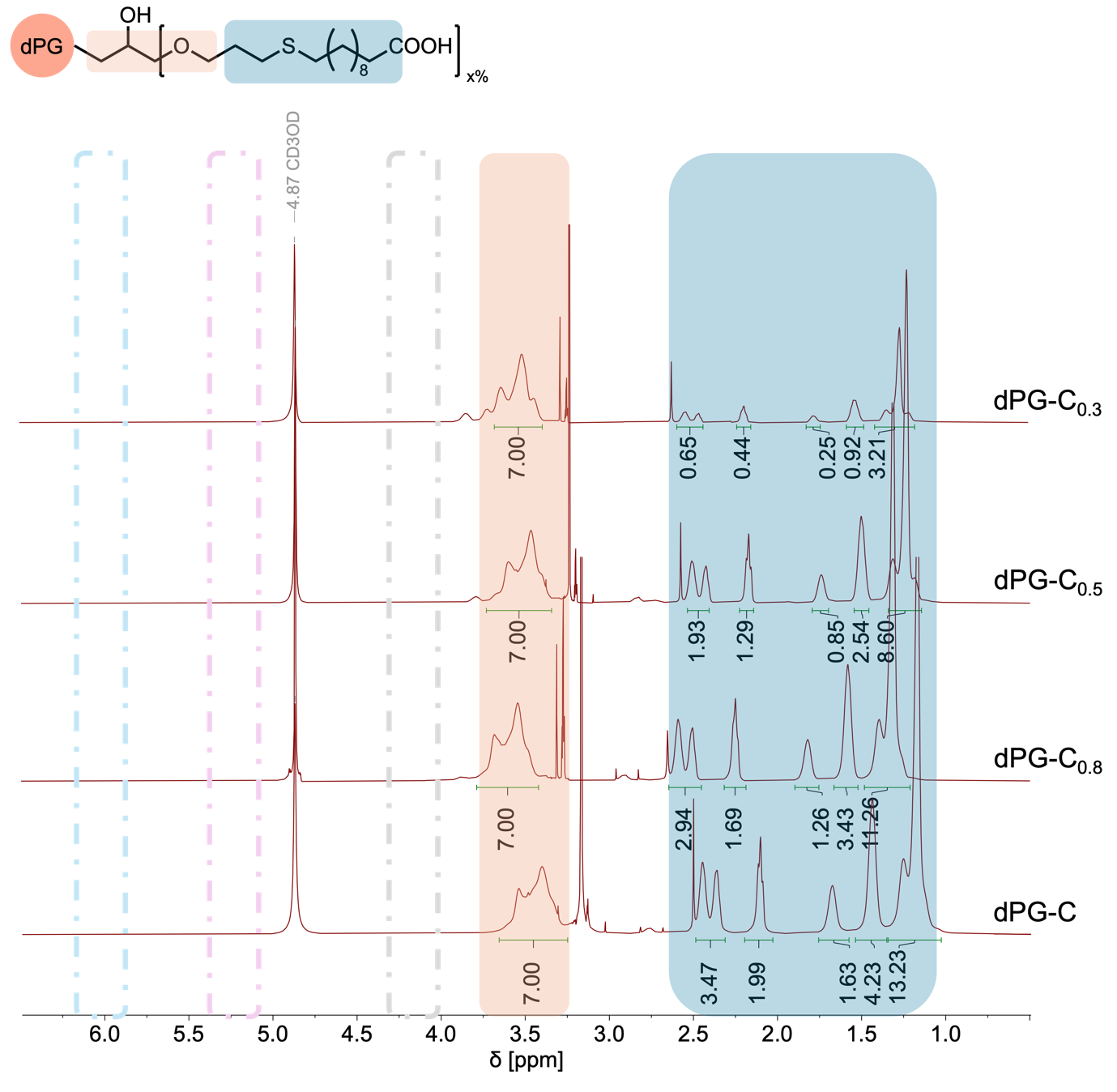


Figure S4. ^1^H-NMR spectra and signal assignment of all carboxylated materials_._ All allylic groups were converted to carboxylic moieties, as indicated by disappearance of the allylic protons signals and the appearance of upfield signals corresponding to the protons of the ligand.

Figure S5. Reaction conditions of the sulfation.

Table S4: reaction scales for the sulfation.

| **DF**  **[%]** | **dPG-MUA** | | | **Py•SO_3_** | | |
| --- | --- | --- | --- | --- | --- | --- |
|  | m  [g] | n_FG_ [mmol] | eq. | m  [g] | n [mmol] | eq. |
| **30** | 0.5 | 3.2 | 1.0 | 2.5 | 16.0 | 5.0 |
| **50** | 0.5 | 2.3 | 1.0 | 1.8 | 11.5 | 5.0 |
| **80** | 0.5 | 1.7 | 1.0 | 1.4 | 8.5 | 5.0 |

Table S5: Elemental analysis of sulfated materials.

|  | **C [%]** | **H [%]** | **N [%]** | **S [%]** |
| --- | --- | --- | --- | --- |
| **dPG-C_0.3_/S** | 36.3 | 6.5 | 0.0 | 12.3 |
| **dPG-C_0.5_/S** | 47.5 | 9.1 | 0.0 | 10.5 |
| **dPG-C_0.8_/S** | 53.6 | 9.6 | 0.0 | 9.3 |

Table S6: Zeta-potential of materials.

|  | $\boldsymbol{\zeta}$ **[mV]** | | | |
| --- | --- | --- | --- | --- |
| **pH** | **7.4** | **7.6** | **7.8** | **8.0** |
| **dPG** | -4 ± 1 | - | - | - |
| **dPG-C_0.3_** | -40 ± 3 | -39 ± 1 | -28 ± 1 | -35 ± 1 |
| **dPG-C_0.3_/S_0.7_** | -27 ± 2 | -26 ± 2 | -29 ± 2 | -27 ± 1 |
| **dPG-C_0.5_** | -38 ± 3 | -35 ± 1 | -29 ± 2 | -28 ± 1 |
| **dPG-C_0.5_/S_0.5_** | -29 ± 2 | -27 ± 2 | -29 ± 1 | 28 ± 3 |
| **dPG-C_0.8_** | -35 ± 1 | -29 ± 2 | -13 ± 1 | -22 ± 2 |
| **dPG-C_0.8_/S_o.2_** | -25 ± 3 | -23 ± 1 | -19 ± 2 | -21 ± 2 |
| **dPG-C** | -38 ± 2 | -37 ± 3 | -32 ± 1 | -3 ± 2 |

Table S7: DLS results of materials.

|  | **Size [nm]** | | | |
| --- | --- | --- | --- | --- |
| **pH** | **7.4** | **7.6** | **7.8** | **8.0** |
| **dPG** | 249 ± 32 | - | - | - |
| **dPG-C_0.3_** | 272 ± 72 | 124 ± 3 | 137 ± 1 | 100 ± 1 |
| **dPG-C_0.3_/S_0.7_** | 191 ± 2 | 196 ± 2 | 196 ± 1 | 209 ± 3 |
| **dPG-C_0.5_** | 153 ± 3 | 171 ± 2 | 95 ± 1 | 68 ± 2 |
| **dPG-C_0.5_/S_0.5_** | 102 ± 2 | 98 ± 1 | 97 ± 2 | 95 ± 3 |
| **dPG-C_0.8_** | 157 ± 2 | 78 ± 1 | 44 ± 1 | 33 ± 1 |
| **dPG-C_0.8_/S_o.2_** | 48 ± 1 | 36 ± 1 | 37 ± 1 | 39 ± 1 |
| **dPG-C** | 162 ± 1 | 133 ± 6 | 80 ± 4 | 29 ± 2 |


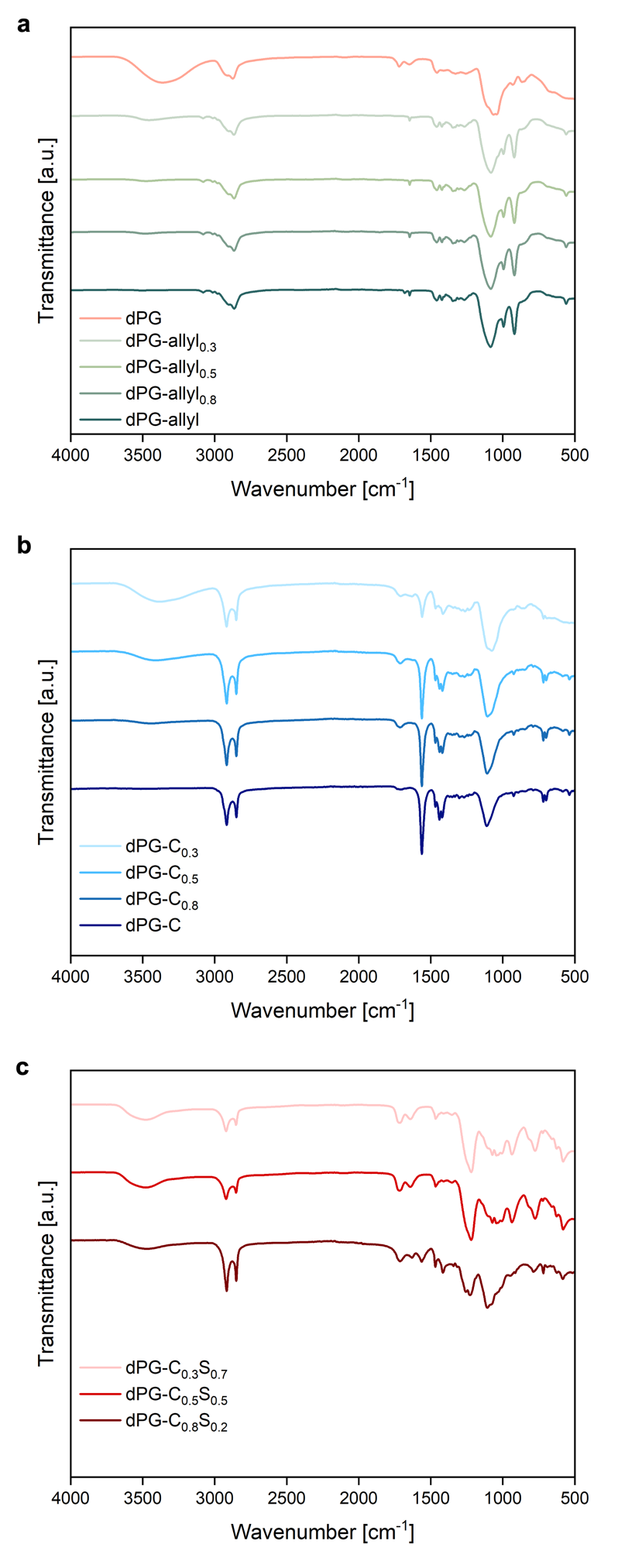


Figure S6. FTIR data for (a) allylated (b) carboxylated and (c) sulfated material.


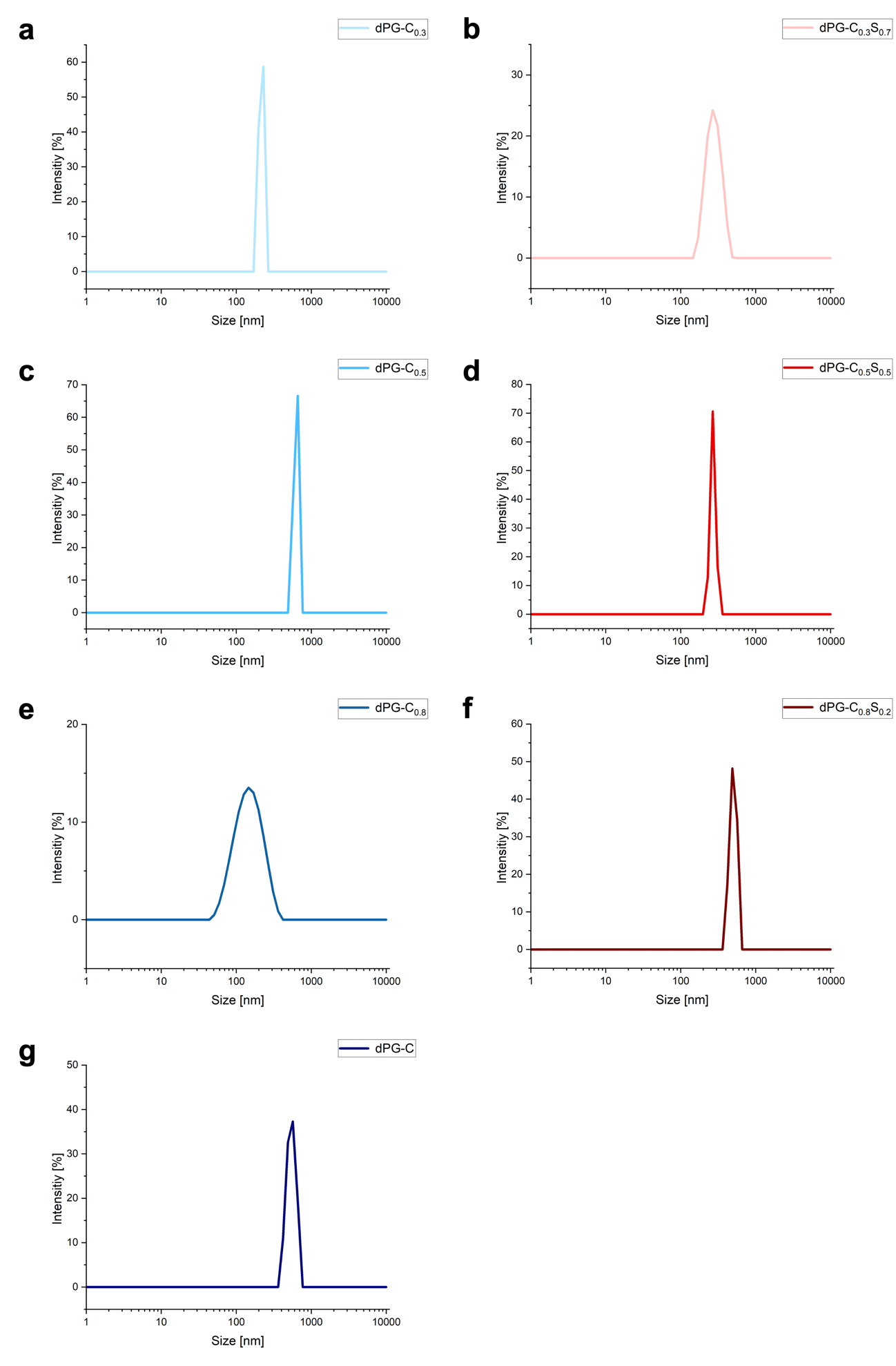


Figure 7. Size distribution curves expressed in terms of the intensity of the scattered light for all materials dissolved in PBS.


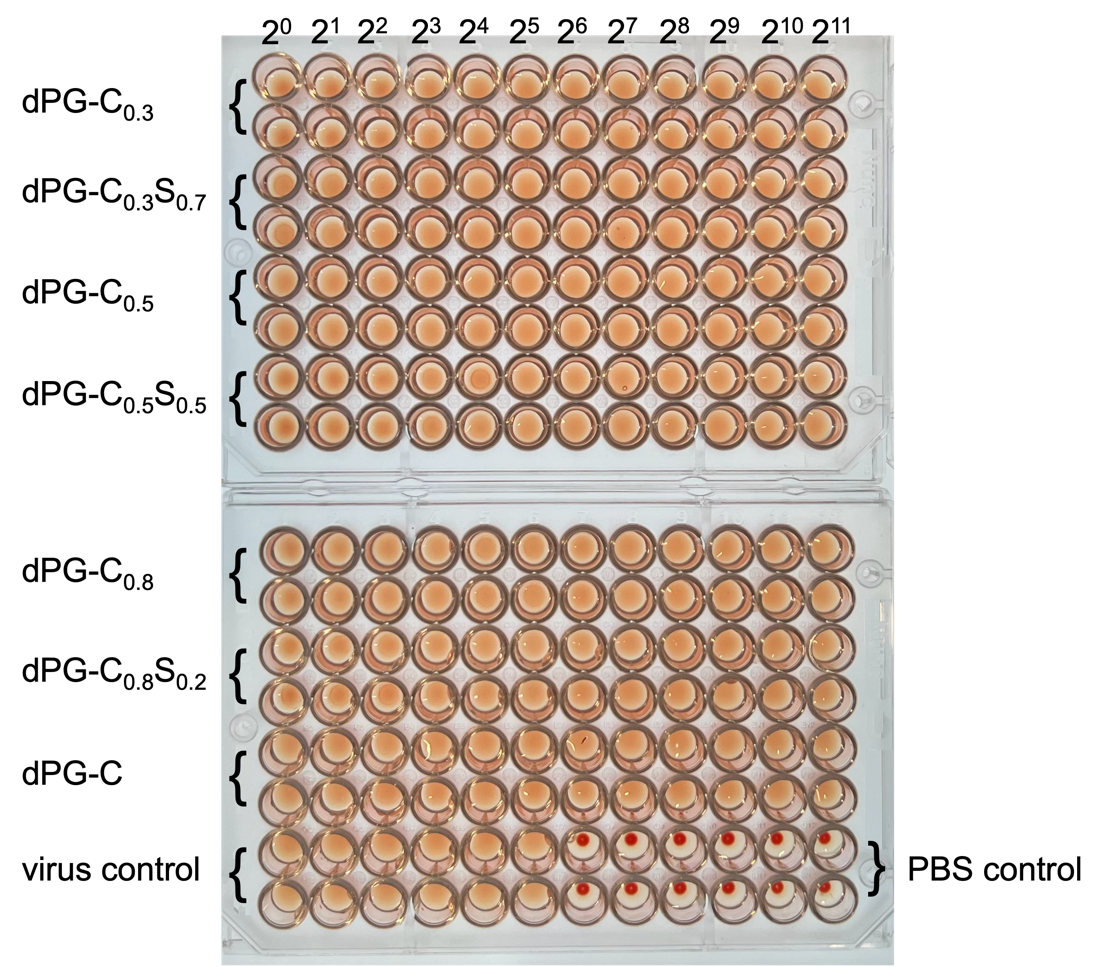


Figure S8. HAI assay. Samples were diluted 2-foldly in 25 µL, incubated with IAV and then treated with RBC. All samples display hemagglutination, which indicates that samples do not bind on HA of IAV.


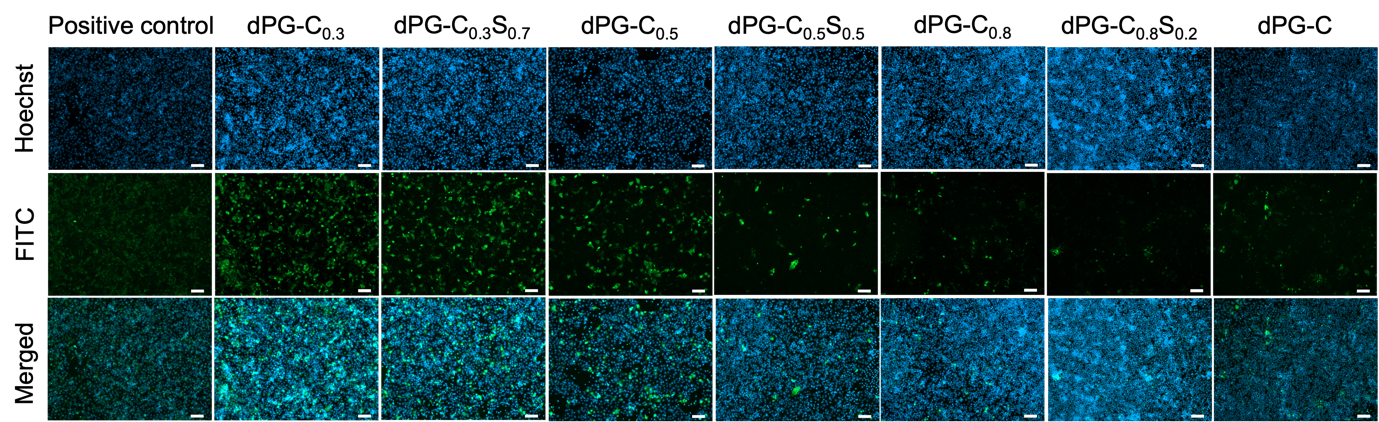


Figure S9. IAV pre-infection assay. Cell nuclei were stained with Hoechst (blue) while infected cells were stained by antibodies with FITC (green). The depicted concentration is 500 µg/mL. The scale bar presents 100 µm.


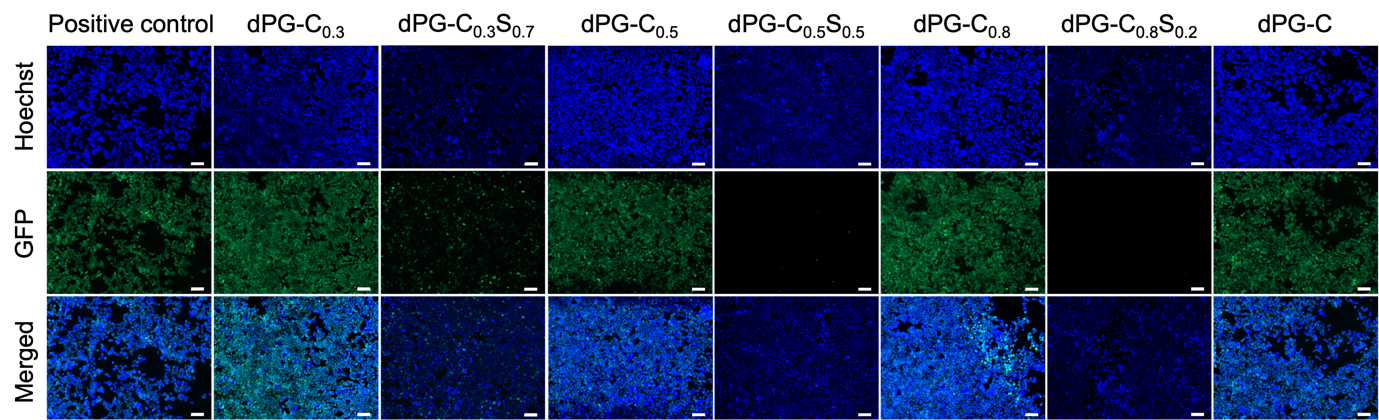


Figure S10. HSV-1 pre-infection assay. Cell nuclei were stained with Hoechst (blue) while infected cells express GFP (green). The depicted concentration is 1 µg/mL. The scale bar presents 100 µm.


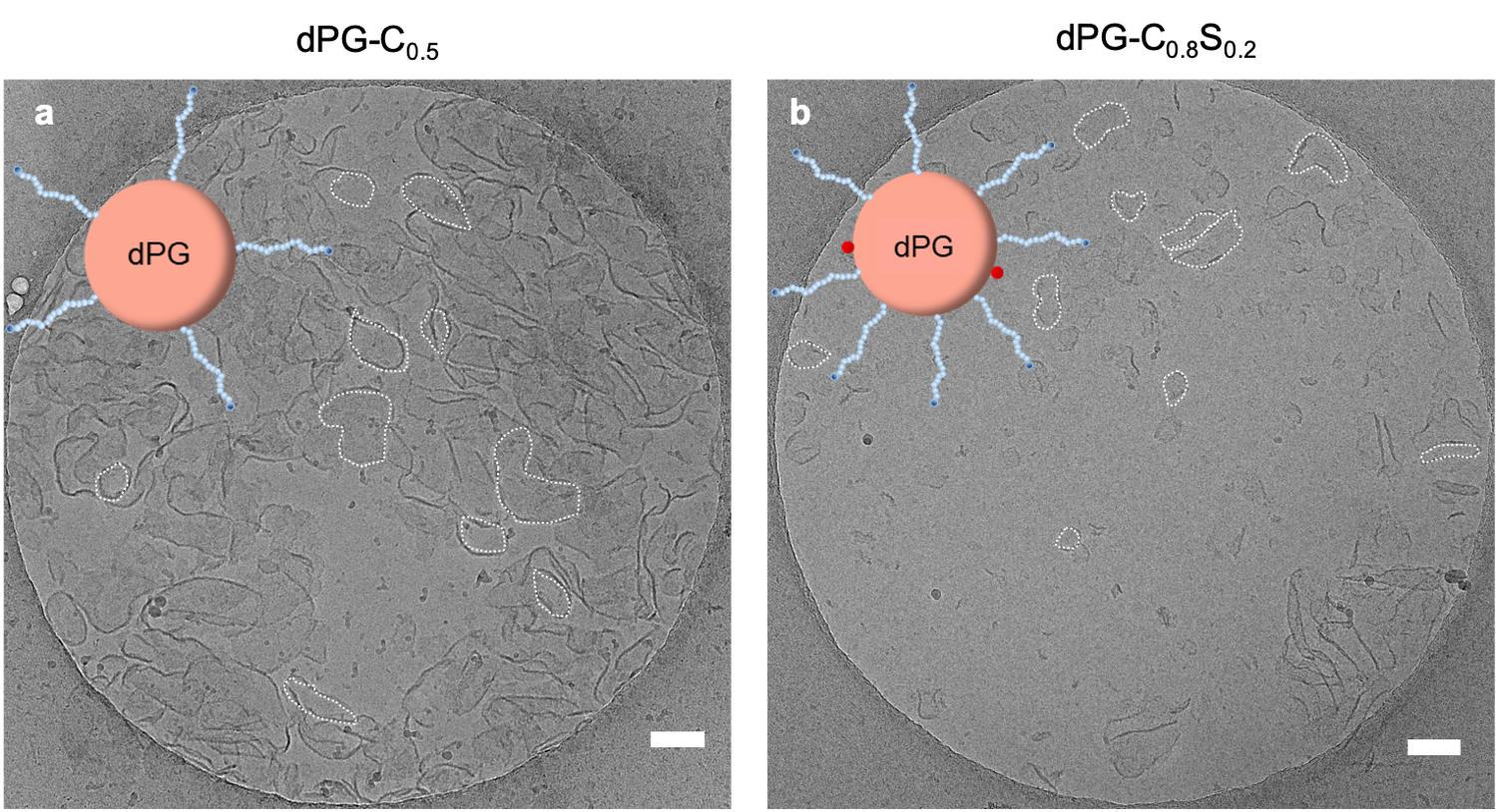
Figure S11. Highlighted edges of sheets in cryo-TEM images for a) dPG-C_0.5_ and b) dPG-C_0.8_S_0.2_. The scale bars corresponding to 100 nm.

_
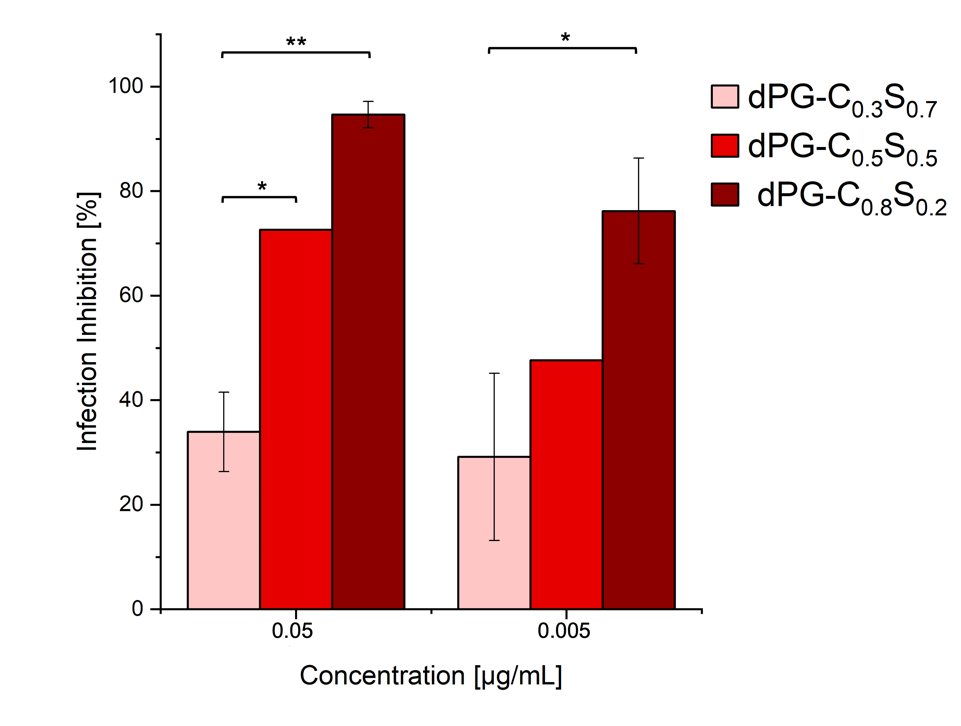
_

Figure S12. Statistical analysis of HSV-1 plaque reduction assay. The data is presented as mean ± SD. The data were evaluated by one-way ANOVA following Tukey’s test using GraphPad Prism software. NS, *, **,and *** represent no signiﬁcant diﬀerence, P > 0.05, p ≤ 0.05, p < 0.01, and p < 0.001, respectively.
